# Diagnostic accuracy of physical examination for the assessment of popliteal cysts: a meta-analysis

**DOI:** 10.3389/fmed.2026.1797462

**Published:** 2026-03-19

**Authors:** Lu Tang, Meng Liu, Bin Sheng, Liping Hu, Qingjiao Li, Hua Cai

**Affiliations:** 1 Department of Interventional Vascular Surgery, Hunan Provincial People’s Hospital, The First-Affiliated Hospital of Hunan Normal University, Changsha, Hunan, China; 2 School of Nursing, Xiangnan University, Chenzhou, China; 3 Department of Orthopedics, Hunan Provincial People’s Hospital, The First-Affiliated Hospital of Hunan Normal University, Changsha, Hunan, China; 4 Department of Intensive Care Medicine, Hunan Provincial People’s Hospital, The First-Affiliated Hospital of Hunan Normal University, Changsha, Hunan, China; 5 Department of Rehabilitation, Hunan Provincial People’s Hospital, The First-Affiliated Hospital of Hunan Normal University, Changsha, Hunan, China; 6 Department of Clinical Nutrition, Hunan Provincial People’s Hospital, The First-Affiliated Hospital of Hunan Normal University, Changsha, Hunan, China

**Keywords:** diagnostic accuracy, meta-analysis, MRI, physical examination, popliteal cysts, ultrasound

## Abstract

**Background:**

Popliteal cysts are common knee cystic lesions and a potential risk factor for knee osteoarthritis, but the vast majority are asymptomatic. Early diagnosis is crucial for effective management. Physical examination is a convenient, non-invasive, and cost-free diagnostic tool, but its accuracy for popliteal cysts remains controversial. This meta-analysis evaluates the diagnostic value of physical examination for popliteal cysts.

**Methods:**

PubMed, Embase, Web of Science, China National Knowledge Infrastructure, and Wanfang databases were searched for studies on physical examination for popliteal cyst diagnosis from inception to November 30, 2025. Study quality was assessed using Quality Assessment of Diagnostic Accuracy Studies-2 (QUADAS-2). Heterogeneity was evaluated using the *I*^2^ index. A bivariate random-effects model calculated pooled sensitivity, specificity, positive likelihood ratio, negative likelihood ratio, diagnostic odds ratio, and 95% confidence intervals (95% CI). Summary receiver operating characteristic curves and area under the curve were analyzed. Sensitivity analysis assessed result robustness.

**Results:**

Ten studies involving 542 patients (720 knee joints) were included. Physical examination showed an area under the curve of 0.88 (95% CI: 0.85–0.91), sensitivity of 0.74 (95% CI: 0.49–0.89), specificity of 0.88 (95% CI: 0.65–0.97), positive likelihood ratio of 6.3 (95% CI: 2.0–19.5), negative likelihood ratio of 0.30 (95% CI: 0.14–0.62), and diagnostic odds ratio of 21 (95% CI: 5–84). Sensitivity analysis confirmed result stability.

**Conclusion:**

Physical examination demonstrates moderate diagnostic accuracy for popliteal cysts. Given the often asymptomatic nature of small cysts and the low clinical burden, physical examination is recommended as a preliminary screening tool when imaging modalities like ultrasonography are unavailable.

**Level of evidence:**

Level 2, diagnostic meta-analysis based on studies with reference standards.

## Introduction

Popliteal cysts, also known as Baker’s cysts, are common space-occupying cystic lesions located in the popliteal region of the knee joint. Their prevalence ranges from 7 to 37% (1–4). Most patients with popliteal cysts are asymptomatic. However, some patients, particularly those with severe cases, may experience symptoms such as knee pain, palpable swelling or mass in the popliteal region, and restricted mobility (5). Compression of blood vessels can lead to localized ischemia or thrombosis, while nerve compression may result in peripheral neuropathy. Therefore, early, rapid, and low-cost diagnosis is essential for improving patients’ quality of life and prognosis (6–10).

Clinically, the recognized methods for the definitive diagnosis of popliteal cysts include pathological diagnosis, X-ray (arthrography), magnetic resonance imaging (MRI), and ultrasound examinations (11). Pathological diagnosis is considered the gold standard for diagnosing popliteal cysts, although it requires invasive procedures such as biopsy or surgical excision. X-ray is the simplest and most accessible method but generally only reveals associated pathologies of the popliteal cysts. Arthrography is rarely used in clinical practice due to its invasive nature, which may exacerbate the condition or irritate surrounding tissues, leading to new symptoms. MRI has limited availability, is time-consuming, and expensive, making it infrequently utilized as the initial examination method for patients with popliteal cysts. Similarly, ultrasound examination depends on equipment and requires a skilled operator, and it also involves costs and waiting time (12, 13). Although the prevalence of popliteal cysts is high, the majority of patients experience mild symptoms, and the primary treatment approach is conservative management (14–16). Therefore, there is a clinical need for a diagnostic method that is simple, convenient, quick, and cost-effective for diagnosing popliteal cysts.

Physical examination (PE), including assessment of Foucher’s sign and simple palpation, has previously been proposed as a potential method for diagnosing popliteal cysts and is routinely used in clinical practice. However, the effectiveness of PE in the diagnosis of popliteal cyst is still controversial (17–26). There is also a lack of relevant evidence from evidence-based medicine worldwide. Therefore, this study aims to conduct a meta-analysis for the first time to evaluate the diagnostic value of PE compared to reference standards (such as X-ray, MRI, and ultrasound) for popliteal cysts, with the goal of providing evidence-based support for the clinical diagnosis and decision-making related to popliteal cysts.

## Methods

### Protocol

This study was conducted in accordance with the preferred reporting items for systematic review and meta-analysis of diagnostic test accuracy studies (PRISMA-DTA) reporting guidelines (27).

### Literature search

PubMed, Embase, Web of Science, China National Knowledge Infrastructure (CNKI) and Wanfang Database were searched for articles on PE for diagnosis of popliteal cyst published from the beginning of the database establishment to November 30, 2025. The search included both Chinese and English publications, focusing on obtaining original articles, without language restrictions. Keywords and Medical Subject Headings terms were appropriately refined and adapted to meet the specific search and syntax requirements of these databases (refer to Supplementary Data 1 for the complete electronic search strategy). The reference lists of included studies were manually reviewed to identify additional relevant studies. Two reviewers independently screened the data sources, and any disagreements were resolved through consultation with a third reviewer (HC).

### Inclusion/exclusion criteria

Articles meeting the following criteria were included: (1) any literature that uses PE compared to X-ray (arthrography), MRI, or ultrasound to diagnose popliteal cysts; (2) studies that provide extractable data for constructing a 2 × 2 contingency table based on reference standards for the diagnosis of popliteal cysts using PE; (3) no language restrictions.

The exclusion criteria were as follows: (1) studies involving cadaveric subjects or non-human research; (2) duplicate publications (selecting the study with the largest dataset); (3) reviews; (4) abstract or title publication only.

### Data extraction

Two researchers independently screened the literature (including screening entries obtained from databases and manually reviewing all references within included studies), extracted data, and cross-checked the information. In cases of disagreement, a discussion was held to reach a consensus. If no agreement could be reached, a third expert was consulted to make the final decision.

The extracted data included: (1) basic information about the included studies, such as study title, authors, publication year, and study type; (2) participant characteristics, including age, number of male and female patients, and associated conditions; (3) key diagnostic parameters, including detailed methods of PE and data for constructing the 2 × 2 contingency table; (4) information related to the quality assessment of the studies.

### Quality assessment

Two researchers independently evaluated the quality of the included studies using the tool of Quality Assessment of Diagnostic Accuracy Studies-2 (QUADAS-2). The results were cross-checked, and in case of disagreements, a discussion was held to reach a consensus. If a resolution could not be achieved, a third expert was consulted to make the final decision (28). The quality of the literature was assessed using QUADAS-2, focusing on two aspects: the risk of bias and clinical applicability. The evaluation results were categorized as high risk, unclear risk, or low risk. The risk of bias graph was generated using Review Manager 5.4 software, presenting the final quality assessment results.

### Statistical analysis

To evaluate the effectiveness of PE for diagnosis, a bivariate random-effects model was employed to calculate the pooled sensitivity, specificity, positive likelihood ratio (PLR), negative likelihood ratio (NLR), diagnostic odds ratio (DOR), and the corresponding 95% confidence intervals (95% CI). Additionally, the summary receiver operating characteristic (SROC) curve was plotted to determine the area under the curve (AUC) size (29). The inconsistency index (*I*^2^) statistic was used to assess heterogeneity, with the following interpretation: 0 to 40% indicates low heterogeneity; 30 to 60% indicates moderate heterogeneity; 50 to 90% indicates substantial heterogeneity; and 75 to 100% indicates considerable heterogeneity (30). To assess the robustness of the pooled estimates, four sensitivity analyses were conducted: (1) exclusion of low-quality studies; (2) exclusion of small-sample studies; (3) exclusion of the three studies that did not explicitly report participant source and age characteristics; (4) exclusion of studies in which palpation was used as a standalone test; and (5) leave-one-out analysis. In the leave-one-out analysis, each study was sequentially omitted and the pooled effect size was recalculated to examine its influence on the overall estimates and between-study heterogeneity (11).

All statistical analyses were conducted using STATA software (V.15.0, Stata, College Station, Texas, United States) and/or Review Manager 5.4 software (RevMan 5.4, The Cochrane Collaboration, Oxford, United Kingdom).

## Results

### Literature search

**Figure 1:**
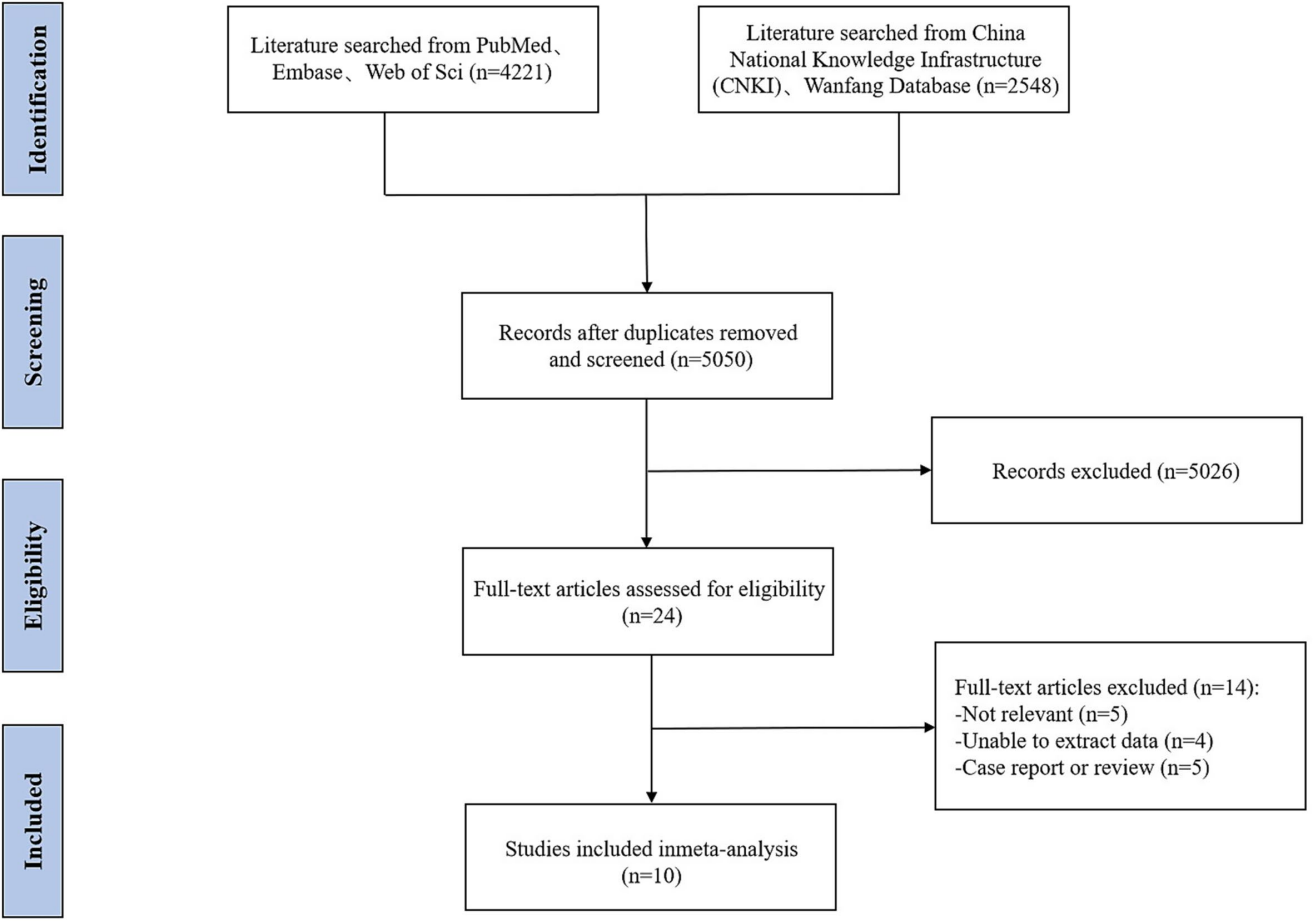
Flowchart of the literature search and screening process.

6,769 articles were retrieved from the databases using standard search strategies, including 4,221 English articles and 2,548 Chinese articles. After merging and removing duplicates, 5,050 articles remained. By reviewing the titles and abstracts, 24 articles were initially screened out as irrelevant. Further full-text reviews were conducted, and based on the pre-established inclusion and exclusion criteria, a total of 10 articles were ultimately included in the meta-analysis (17–26). The detailed literature screening process is shown in Figure 1.

### Basic characteristics and quality assessment

A total of 10 studies were included, including 542 patients (720 knee joints). The study population included both children and older people. Regarding participant sources, two studies enrolled patients with clinically suspected popliteal cysts; one included patients with a unilateral popliteal mass; two enrolled patients with rheumatoid arthritis; one included patients with significant knee pain and functional impairment; and one enrolled patients with knee osteoarthritis. Three studies did not clearly report participant inclusion characteristics.

**Figure 2:**
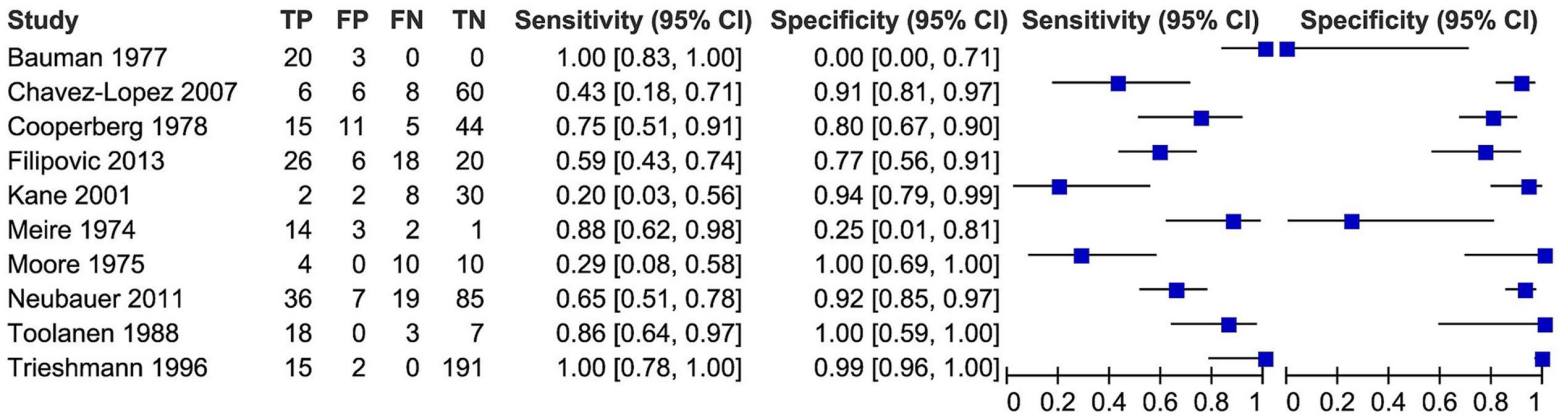
The paired forest plots for the diagnostic accuracy of examination. TP, True positive; FP, False positive; FN, False negative; TN, True negative; CI, Confidence intervals.

Seven studies used ultrasound as the reference standard for diagnosing popliteal cyst. One study used ultrasound and X-ray (arthrography) as the reference standard for diagnosing popliteal cyst. One study used X-ray (arthrography) as the reference standard for diagnosing popliteal cyst. Another study used MRI as the reference standard for diagnosing popliteal cysts. The basic information of the included studies is detailed in Table 1. The extracted original four-fold table data and the forest plot of diagnostic efficacy for each study are detailed in Figure 2.

**Table 1:** Characteristics of included studies.

First author	Year	Patients (*n*)	Knees (*n*)	Participant characteristics	Age (mean years)	Females (*n*)	Country	Reference test
Bauman (17)	1977	19	23	Clinically suspected popliteal cyst	49	15	Germany	Arthrography
Alfredo Chavez-Lopez (18)	2007	40	80	Rheumatoid arthritis patients	61.3	32	Spain	Ultrasound
Cooperberg (19)	1978	38	75	NA	NA	NA	Canada	Ultrasound
Filipovic (20)	2013	70	70	Knee osteoarthritis patients	57.8	49	Serbia	Ultrasound
Kane (21)	2001	22	42	Rheumatoid arthritis patients	50.2	20	United States	Ultrasound
Meire (22)	1974	20	23	NA	NA	NA	Britain	Ultrasound and Arthrography
Moore (23)	1975	17	24	NA	NA	NA	United States	Ultrasound
Neubauer (24)	2011	80	147	Clinically suspected popliteal cyst	8.6	34	Germany	Ultrasound
Toolanen (25)	1988	28	28	History of unilateral popliteal mass	40	8	Sweden	Ultrasound
Trieshmann (26)	1996	208	208	Significant knee pain and dysfunction	45	103	United States	Nuclear magnetic resonance

NA, not available.

The QUADAS-2 quality assessment results indicate that the quality of the 10 included studies is relatively low (Supplementary Figures S1, S2). The primary reason for this is that many of the included studies were published long ago and did not adhere to standard writing formats, resulting in a significant amount of missing information.

### The combined diagnostic efficacy of clinical physical examination

**Figure 3:**
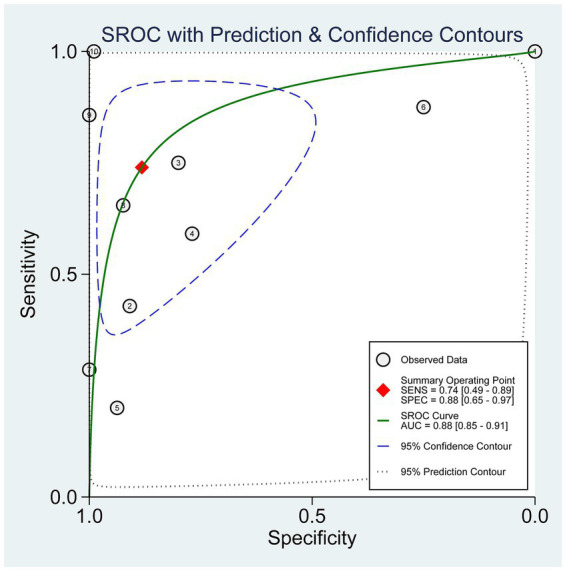
SROC curve of clinical physical examination compared to reference standards for the diagnosis of popliteal cysts. SENS, sensitivity; SPEC, specificity; AUC, area under the curve.

The pooled diagnostic accuracy of PE compared with the reference standard for diagnosing popliteal cysts was as follows: the combined AUC was 0.88 (95% CI: 0.85–0.91, *I*^2^ = 93), sensitivity was 0.74 (95% CI: 0.49–0.89, *I*^2^ = 82), specificity was 0.88 (95% CI: 0.65–0.97, *I*^2^ = 88), PLR was 6.3 (95% CI: 2.0–19.5, *I*^2^ = 92), NLR was 0.30 (95% CI: 0.14–0.62, *I*^2^ = 88), and DOR was 21 (95% CI: 5–84, *I*^2^ = 100) (Table 2 and Figure 2). The SROC curve is presented in Figure 3.

**Table 2:** Diagnostic accuracy of clinical physical examination compared to reference standards for popliteal cyst.

AUC (95% CI)	Sensitivity (95% CI)	Specificity (95% CI)	PLR (95% CI)	NLR (95% CI)	DOR (95% CI)
0.88 (0.85, 0.91)	0.74 (0.49, 0.89)	0.88 (0.65, 0.97)	6.3 (2.0, 19.5)	0.30 (0.14, 0.62)	21 (5, 84)

AUC, area under the curve; PLR, positive likelihood ratio; NLR, negative likelihood ratio; DOR, diagnostic odds ratio; CI, confidence intervals.

### Sensitivity analysis

Sensitivity analysis demonstrated that the results were stable and reliable. The sensitivity analysis results are presented in Table 3: the AUC ranged from 0.84 to 0.91, the sensitivity values ranged from 0.67 to 0.78, and the specificity values ranged from 0.83 to 0.92. After excluding studies that included children, the combined results remained stable and reliable: the AUC was 0.88 (95% CI: 0.85–0.91); the sensitivity was 0.76 (95% CI: 0.47–0.92); and the specificity was 0.88 (95% CI: 0.58–0.97). After excluding two low-quality studies and studies with small sample sizes (*n* < 30), the pooled diagnostic performance estimates remained largely unchanged, indicating that the overall conclusions were robust (Supplementary Tables S1, S2). After excluding the three studies that lacked clear reporting of participants’ source and age characteristics, a subgroup analysis was performed, which confirmed that the overall findings remained robust (Supplementary Table S3). A subgroup analysis was conducted after excluding the single study that explicitly used palpation as an independent physical examination method, and the results still demonstrated overall robustness of the findings (Supplementary Table S4). The above subgroup analyses further support the robustness of our findings.

**Table 3:** Sensitivity analysis results.

Excluded studies^*^	AUC (95% CI)	Sensitivity (95% CI)	Specificity (95% CI)
Bauman (17)	0.89 (0.86–0.92)	0.67 (0.46–0.83)	0.92 (0.80–0.97)
Alfredo Chavez-Lopez (18)	0.89 (0.86–0.92)	0.77 (0.51–0.92)	0.88 (0.58–0.97)
Cooperberg (19)	0.89 (0.86–0.91)	0.75 (0.45–0.91)	0.89 (0.62–0.98)
Filipovic (20)	0.89 (0.86–0.91)	0.75 (0.45–0.91)	0.89 (0.62–0.98)
Kane (21)	0.89 (0.85–0.91)	0.78 (0.56–0.91)	0.87 (0.57–0.97)
Meire (22)	0.91 (0.88–0.93)	0.72 (0.44–0.90)	0.91 (0.75–0.97)
Moore (23)	0.89 (0.86–0.91)	0.78 (0.54–0.92)	0.86 (0.61–0.96)
Neubauer (24)	0.88 (0.85–0.91)	0.76 (0.47–0.92)	0.88 (0.58–0.97)
Toolanen (25)	0.88 (0.85–0.91)	0.76 (0.47–0.92)	0.88 (0.58–0.97)
Trieshmann (26)	0.84 (0.80–0.87)	0.70 (0.45–0.87)	0.83 (0.61–0.94)

^*^After excluding one study, the combined results of the remaining studies were analyzed to evaluate the stability of the findings. AUC, area under the curve.

## Discussion

This meta-analysis demonstrated that PE has moderate diagnostic accuracy for popliteal cysts when compared with reference standards such as X-rays, MRI, and ultrasound. Sensitivity analysis confirmed the stability of the results. These findings provide evidence supporting the use of PE as an initial screening tool for popliteal cysts in clinical practice.

Clinically, various methods are available to evaluate and diagnose popliteal cysts, including MRI, X-rays, and ultrasound. Among these, MRI is considered the imaging gold standard for diagnosing popliteal cysts (14, 31). However, MRI is limited by its high cost and difficulty in widespread implementation. The primary role of X-rays is to assess knee joint degeneration and the presence of loose bodies. However, it cannot directly diagnose popliteal cysts, presenting certain limitations. Ultrasound is another imaging method used clinically to evaluate popliteal cysts. It also requires the use of equipment, and ultrasound examination is often dependent on the operator’s technical skill. Therefore, the operator needs to undergo appropriate professional training and assessment.

Besides diagnosing the presence of a popliteal cyst, clinical practice often requires information on the cyst’s size, classification (such as simple type, lobulated type and cystic cloudy type), and whether it communicates with the joint cavity to provide relevant information for clinical treatment (32). Imaging examinations can obtain or partially provide the above information. However, PE is inadequate for acquiring the characteristic features of popliteal cysts. Therefore, PE may primarily serve as a method for initial clinical screening (33).

In clinical practice, popliteal cysts are common, and most patients are either asymptomatic or, if symptomatic, are initially managed with conservative treatment. Therefore, there is an urgent need for a rapid, convenient, low-cost diagnostic method suitable for various settings, such as outpatient clinics and wards (15). It is recommended to combine PE with the patient’s medical history (such as frequent labor, exercise, or related joint injuries and pain) as a screening method for popliteal cysts, which could reduce the examination costs for the patients.

Previously reported PE methods for popliteal cysts primarily include palpation, Foucher’s sign, fluctuation / ballottement tests, hyperextension tenderness test, and knee flexion palpation test. For palpation, one hand is placed behind the popliteal fossa while the patient’s knee is either fully extended or flexed to 90°, and alternating shallow and deep palpation can detect a round, smooth, elastic mass in the central or slightly lateral popliteal region, sometimes with fluctuation (33, 34). Foucher’s sign evaluates cyst tension dynamically during knee flexion and extension: the cyst becomes tense and may be tender on hyperextension, whereas it softens or disappears on flexion (12, 33–35). Fluctuation or ballottement tests assess the cystic nature of the mass with gentle pressure, while hyperextension tenderness and knee flexion palpation further evaluate cyst size, shape, and tenderness (5, 36). These PE methods differ in clinical feasibility. Palpation is simple and rapid but highly operator-dependent, resulting in relatively lower diagnostic accuracy (33, 34, 36). Fluctuation testing can suggest a cystic structure but may miss small or deep cysts (5, 33, 36). The mechanism of Foucher’s sign is mainly related to the approximation of the medial head of the gastrocnemius and the semimembranosus tendon during knee hyperextension, which compresses the cyst and increases intra-cystic pressure. By dynamically evaluating cyst tension, Foucher’s sign can reliably differentiate popliteal cysts from other masses that do not change with knee movement, such as tumors, hemangiomas, or nerve sheath tumors, showing superior specificity and discriminative ability compared to static palpation or fluctuation assessment (12, 37). In clinical practice, multiple PE methods are usually applied in combination, and most studies included in this meta-analysis employed a comprehensive PE approach rather than a single test. Therefore, combining Foucher’s sign, palpation, and other physical examination methods is recommended to improve the diagnostic accuracy of popliteal cysts.

However, it must be acknowledged that the ability of PE to differentiate popliteal cysts may not be as accurate as imaging diagnostic methods, as the clinician’s experience plays a significant role in the diagnosis and differential diagnosis of popliteal cysts. Currently, there is no standardized method for the PE of popliteal cysts, highlighting the need to develop more standardized PE protocols in the future.

This study has several limitations: (1) The heterogeneity between studies was considerable; therefore, we employed a random-effects model for result aggregation to obtain estimates that are closer to the actual values. (2) There is a lack of literature detailing specific methods or techniques for PE in the diagnosis of popliteal cysts. Inaccurate PE techniques may lead to underestimating the diagnostic accuracy of PE for popliteal cysts. Establishing standardized PE protocols may enhance the diagnostic accuracy of PE in identifying popliteal cysts. (3) The small sample size of patients in the included studies may increase the likelihood of random variation in the research findings. (4) Although popliteal cyst is considered a non-specific lesion, previous studies have shown that its typical clinical manifestations, such as popliteal swelling, mild pain, and limited range of motion, are broadly comparable among patients with KOA, RA, and the general population (5). This indicates that physical examination may have relatively consistent diagnostic applicability across different clinical contexts, which is in line with our findings. Nevertheless, the majority of participants included in the present analysis had specific underlying knee disorders. Therefore, caution is warranted when directly generalizing the findings to the general population. (5) Due to the limited number of studies in certain subgroups, we were unable to perform subgroup analyses of physical examination based on different reference standards. (6) Additionally, in clinical practice, physical examination is typically based on a combination of multiple maneuvers (e.g., Foucher’s sign, palpation, and other tests), with findings integrated into an overall clinical judgment rather than relying on a single maneuver. As most included studies used mixed examination approaches and did not report diagnostic accuracy separately, comparison of specific physical examination tests was not feasible. (7) Due to the limited number of eligible studies, we were unable to conduct subgroup analyses according to age categories or underlying disease characteristics of the study populations.

In light of the aforementioned limitations, future research should prioritize large-scale, prospective diagnostic accuracy studies employing standardized physical examination protocols and high-quality imaging reference standards (such as MRI or high-resolution ultrasound) to improve methodological rigor and enhance the reliability of conclusions.

## Conclusion

In summary, clinical PE demonstrates moderate diagnostic accuracy for popliteal cysts. Considering that the majority of patients with popliteal cysts are asymptomatic or have mild symptoms, the clinical burden is relatively low, and conservative treatment is primarily recommended as the initial approach. When imaging techniques such as ultrasound are not readily accessible, PE is recommended as a preliminary screening method for popliteal cysts. However, when there is a need to obtain more detailed information regarding intra-articular lesions, perform accurate differential diagnoses, and provide a reference for surgical decision-making, it is advisable to utilize imaging modalities such as ultrasound and MRI, which can accurately diagnose popliteal cysts and provide additional information.

## Data Availability

The original contributions presented in the study are included in the article/Supplementary material, further inquiries can be directed to the corresponding author.
