# Correction: Diagnostic accuracy of physical examination for the assessment of popliteal cysts: a meta-analysis

**DOI:** 10.3389/fmed.2026.1835440

**Published:** 2026-04-07

**Authors:** Lu Tang, Meng Liu, Bin Sheng, Liping Hu, Qingjiao Li, Hua Cai

**Affiliations:** 1 Department of Interventional Vascular Surgery, Hunan Provincial People's Hospital, The First-Affiliated Hospital of Hunan Normal University, Changsha, Hunan, China; 2 School of Nursing, Xiangnan University, Chenzhou, China; 3 Department of Orthopedics, Hunan Provincial People's Hospital, The First-Affiliated Hospital of Hunan Normal University, Changsha, Hunan, China; 4 Department of Intensive Care Medicine, Hunan Provincial People's Hospital, The First-Affiliated Hospital of Hunan Normal University, Changsha, Hunan, China; 5 Department of Rehabilitation, Hunan Provincial People's Hospital, The First-Affiliated Hospital of Hunan Normal University, Changsha, Hunan, China; 6 Department of Clinical Nutrition, Hunan Provincial People's Hospital, The First-Affiliated Hospital of Hunan Normal University, Changsha, Hunan, China

**Keywords:** diagnostic accuracy, meta-analysis, MRI, physical examination, popliteal cysts, ultrasound

Author Meng Liu was erroneously omitted as equal contributing author. “These authors have contributed equally to this work and share first authorship” has been added for authors Lu Tang and Meng Liu.

The original version of this article has been updated.

