# Supplementary material for: Diagnostic accuracy of physical examination for the assessment of popliteal cysts: a meta-analysis
Source: Front Med (Lausanne). 2026 Mar 19;13:1797462. doi: 10.3389/fmed.2026.1797462 (PMC13044020; doi:10.3389/fmed.2026.1797462)
Supplement: Supplementary file 1 [file Supplementary_file_1.docx]

**Supplemental Data 1**

**Search strategy**

**PubMed:**

1.Sensitivity and Specificity[Mesh] OR Diagnosis[mesh] OR diagnos*[tiab] OR sensitiv*[tiab] OR specificit*[tiab] OR distinguish*[tiab] OR differential*[tiab] OR identif*[tiab] OR detect*[tiab] OR accurac*[tiab] OR comparison[tiab] OR false positive[tiab] OR false positives[tiab] OR false negative[tiab] OR false negatives[tiab]

2.Popliteal Cyst[mesh] OR Popliteal Cyst*[tiab] OR Cyst, Popliteal[tiab] OR Cysts, Popliteal[tiab] OR Baker's Cyst*[tiab] OR Baker Cyst*[tiab] OR Cyst, Baker*[tiab] OR Cysts, Baker*[tiab]

3.1 AND 2

**Embase:**

1.'Sensitivity and Specificity'/exp OR 'Diagnosis'/exp OR 'diagnos*':ti,ab OR 'sensitiv*':ti,ab OR 'specificit*':ti,ab OR 'distinguish*':ti,ab OR 'differential*':ti,ab OR 'identif*':ti,ab OR 'detect*':ti,ab OR 'accurac*':ti,ab OR 'comparison':ti,ab OR 'false positive':ti,ab OR 'false positives':ti,ab OR 'false negative':ti,ab OR 'false negatives':ti,ab

2.'popliteal cyst'/exp OR 'popliteal cyst*':ti,ab OR 'cyst, popliteal':ti,ab OR 'cysts, popliteal':ti,ab OR 'bakers cyst*':ti,ab OR 'baker cyst*':ti,ab OR 'cyst, baker*':ti,ab OR 'cysts, baker*':ti,ab

3.1 AND 2

**Web of Science:**

1.diagnos* (Topic) OR sensitiv* (Topic) OR specificit* (Topic) OR distinguish* (Topic) OR differential* (Topic) OR identif* (Topic) OR detect* (Topic) OR accurac* (Topic) OR comparison (Topic) OR false positive (Topic) OR false positives (Topic) OR false negative (Topic) OR false negatives (Topic)

2.Popliteal Cyst* (Topic) or Cyst, Popliteal (Topic) or Cysts, Popliteal (Topic) or Baker's Cyst* (Topic) or Baker Cyst* (Topic) or Cyst, Baker* (Topic) or Cysts, Baker* (Topic)

3.1 AND 2

**China National Knowledge Infrastructure (CNKI ):**

1.TKA= ("体格检查" + "体查" + "触诊" + "体检" + "临床检查")

2.TKA= ("腘窝" + "baker" + "baker's" + "popliteal" + "贝克")

3.1 AND 2

**Wanfang Database:**

1.全部字段= (体格检查 OR 体查 OR 触诊 OR 体检 OR 临床检查)

2.全部字段=(腘窝 OR Baker OR Baker’s OR Popliteal OR 贝克)

3.1 AND 2


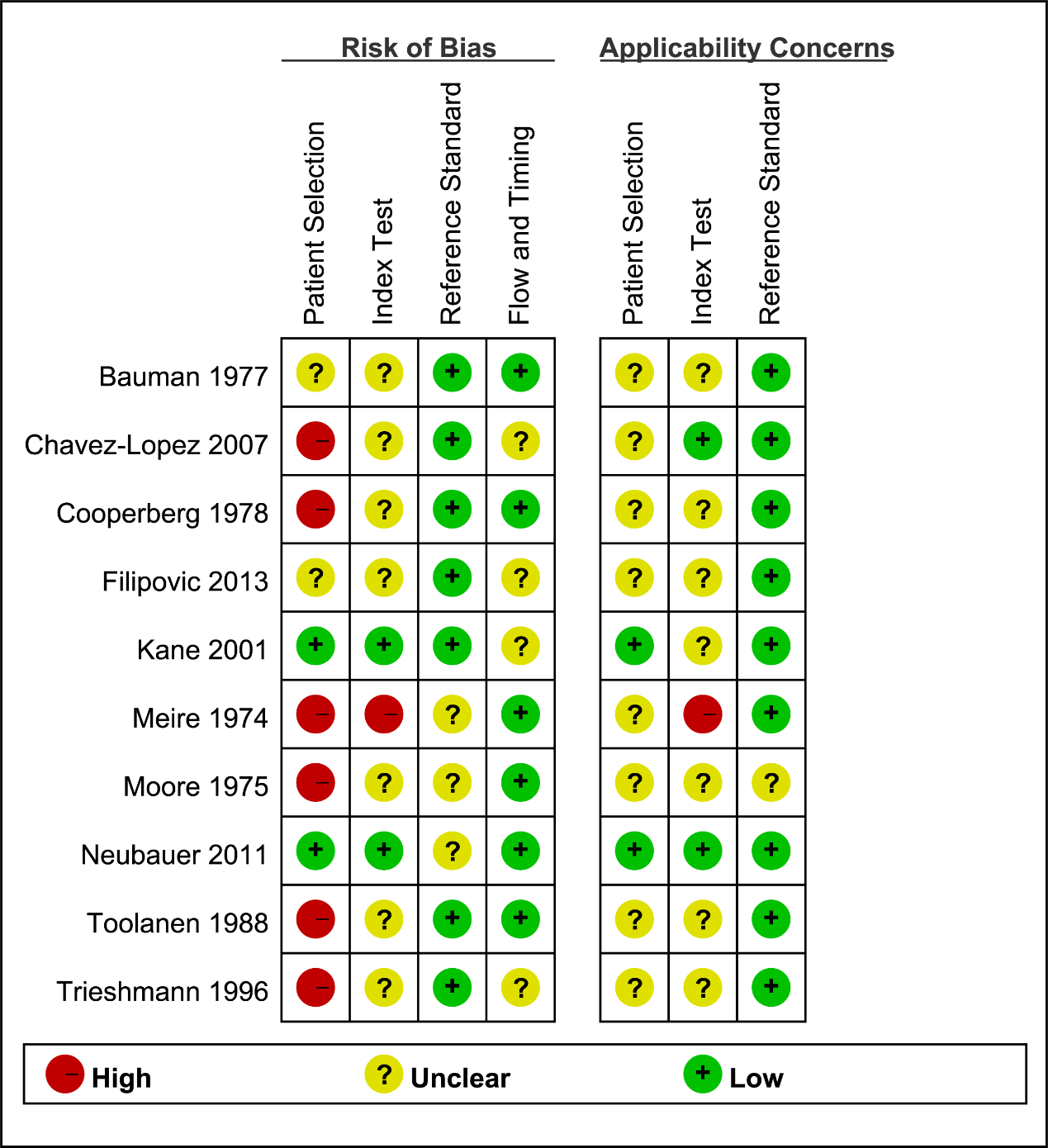


**Supplemental Figure S1. The Cochrane risk of bias and applicability concerns summary.**

**
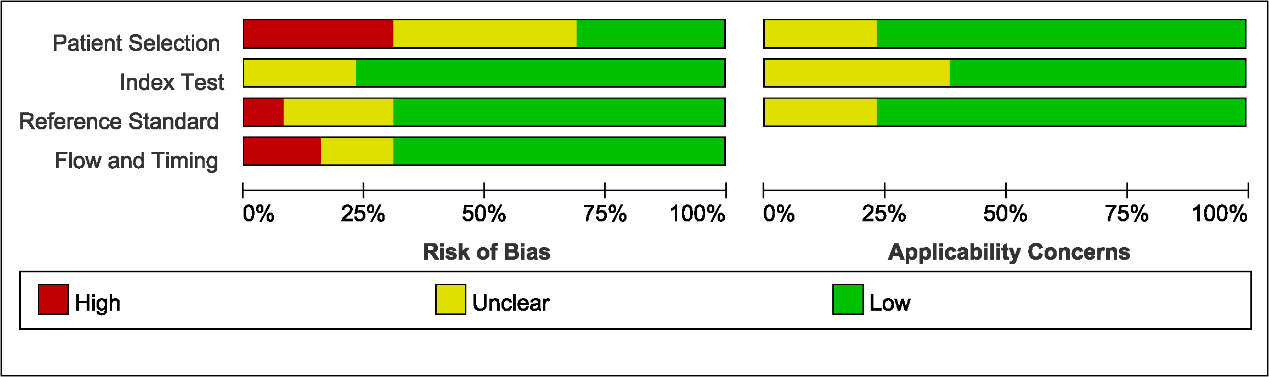
**

**Supplemental Figure S2. The Cochrane risk of bias graph.**

**Supplemental Table S1. Diagnostic accuracy of clinical physical examination for popliteal cysts compared with reference standards after excluding low-quality studies**

| **AUC**  **(95% *CI*)** | **Sensitivity (95% CI)** | **Specificity (95% CI)** | **PLR**  **(95% CI)** | **NLR**  **(95% CI)** | **DOR**  **(95% CI)** |
| --- | --- | --- | --- | --- | --- |
| 0.91  (0.88, 0.93) | 0.77  (0.48, 0.92) | 0.90  (0.72, 0.97) | 7.4  (2.5, 21.7) | 0.26  (0.10, 0.68) | 29  (6, 142) |

AUC, area under the curve; PLR, positive likelihood ratio; NLR, negative likelihood ratio; DOR, diagnostic odds ratio; CI, confidence intervals.

**Supplemental Table S2. Diagnostic accuracy of clinical physical examination for detecting popliteal cysts compared with reference standards after exclusion of small-sample studies**

| **AUC**  **(95% *CI*)** | **Sensitivity (95% CI)** | **Specificity (95% CI)** | **PLR**  **(95% CI)** | **NLR**  **(95% CI)** | **DOR**  **(95% CI)** |
| --- | --- | --- | --- | --- | --- |
| 0.90  (0.87, 0.93) | 0.72  (0.49, 0.87) | 0.92  (0.80, 0.97) | 8.7  (2.7, 27.9) | 0.31  (0.14, 0.66) | 29  （4, 184) |

AUC, area under the curve; PLR, positive likelihood ratio; NLR, negative likelihood ratio; DOR, diagnostic odds ratio; CI, confidence intervals.

**Supplemental Table S3. Diagnostic accuracy of clinical physical examination for popliteal cysts compared with reference standards after excluding studies with unclear participant characteristics**

| **AUC**  **(95% *CI*)** | **Sensitivity (95% CI)** | **Specificity (95% CI)** | **PLR**  **(95% CI)** | **NLR**  **(95% CI)** | **DOR**  **(95% CI)** |
| --- | --- | --- | --- | --- | --- |
| 0.92  (0.90, 0.94) | 0.79  (0.42, 0.95) | 0.91  (0.69, 0.98) | 8.4  (2.2, 31.3) | 0.24  (0.07, 0.82) | 35  （5, 253) |

AUC, area under the curve; PLR, positive likelihood ratio; NLR, negative likelihood ratio; DOR, diagnostic odds ratio; CI, confidence intervals.

**Supplemental Table S4. Diagnostic accuracy of clinical physical examination for popliteal cysts compared with reference standards after excluding studies using palpation as a standalone test**

| **AUC**  **(95% *CI*)** | **Sensitivity (95% CI)** | **Specificity (95% CI)** | **PLR**  **(95% CI)** | **NLR**  **(95% CI)** | **DOR**  **(95% CI)** |
| --- | --- | --- | --- | --- | --- |
| 0.89  (0.85, 0.91) | 0.78  (0.56, 0.91) | 0.87  (0.57, 0.97) | 6.1  (1.6, 23.5) | 0.25  (0.12, 0.54) | 24  （5, 116) |

AUC, area under the curve; PLR, positive likelihood ratio; NLR, negative likelihood ratio; DOR, diagnostic odds ratio; CI, confidence intervals.
